# Liquidity connectedness in cryptocurrency market

**DOI:** 10.1186/s40854-021-00308-3

**Published:** 2022-01-05

**Authors:** Mudassar Hasan, Muhammad Abubakr Naeem, Muhammad Arif, Syed Jawad Hussain Shahzad, Xuan Vinh Vo

**Affiliations:** 1 Lahore Business School, The University of Lahore, Lahore, Pakistan; 2 Smurfit Graduate School of Business, University College Dublin, Dublin, Ireland; 3 Institute of Business Research, University of Economics Ho Chi Minh City, Ho Chi Minh City, Vietnam; 4 Department of Business Administration, Shaheed Benazir Bhutto University, Shaheed Benazirabad, Pakistan; 5 Montpellier Business School, Montpellier, France; 6 South Ural State University, Chelyabinsk, Russian Federation; 7 Institute of Business Research and CFVG, University of Economics Ho Chi Minh City, Ho Chi Minh City, Vietnam

**Keywords:** Liquidity, Time–frequency connectedness, Cryptocurrencies, C10, C32, G01, G15

## Abstract

We examine the dynamics of liquidity connectedness in the cryptocurrency market. We use the connectedness models of Diebold and Yilmaz (Int J Forecast 28(1):57–66, 2012) and Baruník and Křehlík (J Financ Econom 16(2):271–296, 2018) on a sample of six major cryptocurrencies, namely, Bitcoin (BTC), Litecoin (LTC), Ethereum (ETH), Ripple (XRP), Monero (XMR), and Dash. Our static analysis reveals a moderate liquidity connectedness among our sample cryptocurrencies, whereas BTC and LTC play a significant role in connectedness magnitude. A distinct liquidity cluster is observed for BTC, LTC, and XRP, and ETH, XMR, and Dash also form another distinct liquidity cluster. The frequency domain analysis reveals that liquidity connectedness is more pronounced in the short-run time horizon than the medium- and long-run time horizons. In the short run, BTC, LTC, and XRP are the leading contributor to liquidity shocks, whereas, in the long run, ETH assumes this role. Compared with the medium term, a tight liquidity clustering is found in the short and long terms. The time-varying analysis indicates that liquidity connectedness in the cryptocurrency market increases over time, pointing to the possible effect of rising demand and higher acceptability for this unique asset. Furthermore, more pronounced liquidity connectedness patterns are observed over the short and long run, reinforcing that liquidity connectedness in the cryptocurrency market is a phenomenon dependent on the time–frequency connectedness.

## Introduction

Liquidity is a crucial facet of today’s financial markets that encompasses ease, speed, and affordability that an investor can trade. Liquidity is of great relevance to investors and policymakers, as a systematic liquidity factor exists in many financial markets (Chordia et al. 2001; Marshall et al. 2013). Liquidity levels are connected across similar assets and vary over time (Hasbrouck and Seppi 2001). An asset’s liquidity is also linked to market-wide liquidity—an idea often known as liquidity commonality (Chordia et al. 2000; Chuliá et al. 2020). Inekwe (2020) recently introduced liquidity connectedness, which has comprehensively accounted for cross-asset liquidity linkages and liquidity commonality. This network-based approach provides a holistic view of liquidity transmission by identifying the transmitters and receivers of liquidity shocks within a system. Although liquidity commonality has been studied for various financial markets,^1^ until now, liquidity connectedness has only been examined for stock markets (Inekwe 2020).

We examine the liquidity connectedness in the cryptocurrency market. Cryptocurrencies have shown tremendous potential recently; thus, trading volumes in cryptocurrency markets are rising, indicating that the liquidity levels in these markets^2^ are significantly improving (Shahzad et al. 2019). Additionally, the sentiment toward the cryptocurrency market is showing positive signs (Naeem et al. 2020b, 2021b). However, greater institutional involvement means improved liquidity in the cryptocurrency market. This notion implies a heightened risk of liquidity transmission across cryptocurrencies. Massive price swings have brought about sudden and synchronized movements in cryptocurrency liquidity over the past few years, raising serious concerns among investors and policymakers (Al-Yahyaee et al. 2020). Moreover, cryptocurrencies are tightly interlinked (Antonakakis et al. 2019). Thus, trading in cryptocurrencies depends on trading cost (Shahzad et al. 2021a,b), how crypto liquidity links to one another, and market-wide liquidity. Understanding liquidity connectedness in the cryptocurrency market can also help devise trading, investing, and hedging strategies involving cryptocurrencies (Hu et al. 2019) as liquidity is an essential factor in such matters.

The existing literature proposed two potential channels of liquidity connectedness. Demand-induced connectedness can be associated with the synchronized trading behavior of the investors (Chordia et al. 2000; Hasbrouck and Seppi 2001). This case would take place when large trading orders, mainly placed by institutional investors, put pressure on dealers’ inventory levels, further inducing fluctuations in the liquidity levels and co-movements (Kamara et al. 2008). Koch et al. (2016) suggested that index trading by institutional investors becomes an increasingly important source of demand-generated liquidity connectedness. Shocks caused by mutual funds’ simultaneous traded patterns and substantial trade imbalances also lead to liquidity connectedness.

Conversely, supply-induced liquidity connectedness can be attributed to the financial intermediaries’ funding constraints for providing liquidity (Brunnermeier and Pedersen 2009), particularly during significant market downturns that typically result in a liquidity crunch. Coughenour and Saad (2004) and Hameed et al. (2010) showed that increases in cross-industry liquidity spillovers are induced by significant and negative returns in counterpart industries. Such supply-driven liquidity spillovers in industries partly suggest commonality because the liquidity dry-ups affect the entire market.^3^

Although the two channels are not mutually exclusive and could drive liquidity connectedness, we would expect the demand-side channel to significantly influence the liquidity connectedness in the cryptocurrency market.^4^ This argument is motivated by the recent rise of cryptocurrency demand (Foley et al. 2019). This phenomenon has not only made these markets more liquid but also given rise to potentially synchronized liquidity swings. Notably, cryptocurrency markets are crowded with individual investors and speculators, whose trading activities are often characterized by herd behavior (Vidal-Tomás et al. 2019). Once fueled by herd behavior, investors’ speculative demand for cryptocurrencies (Bouri et al. 2019a; da Gama Silva et al. 2019; Gurdgiev and O'Loughlin 2020) is likely to drive the liquidity connectedness across cryptocurrency markets. Additionally, as highlighted above, institutional investors become involved in cryptocurrency trading owing to the various online trading platforms^5^ catering to the rising institutional demand for trading and hedging purposes (Foley et al. 2019). Institutional investors’ liquidity buildups in the cryptocurrency market also potentially strengthen the demand-side channel of liquidity connectedness through correlated trading. Finally, volatility may also induce liquidity connectedness (Chuliá et al. 2020), which aligns with the theoretical framework proposed by Brunnermeier and Pedersen (2009). According to this framework, higher market volatility contributes to a rise in liquidity connectedness, which results from a decline in liquidity provision available for financial intermediaries.

Based on the theoretical background, we take the cryptocurrency liquidity literature a step ahead and explore liquidity connectedness in cryptocurrency markets. This study mainly contributes by exploring liquidity connectedness in financial markets, such as the stock market (Chuliá et al. 2020; Inekwe 2020). More importantly, we investigate liquidity linkages among cryptocurrencies, adding to the previous works on crypto liquidity and its relationship with price efficiency (Brauneis and Mestel 2018; Naeem et al. 2021a). Accordingly, we implement the connectedness model of Diebold and Yilmaz (2012) and the frequency connectedness model of Baruník and Křehlík (2018) to the widely recognized and most liquid set of cryptocurrencies. The relevance of time–frequency analysis of liquidity connectedness emanates from the fact that investors function at different investment horizons, expressed in various trading frequencies, tools, and strategies (Gençay et al., 2010; Bredin et al. 2017). Consequently, through these trading dynamics, the investor’s time horizons could well be reflected in crypto liquidity and its connectedness.

Our static analysis reveals a moderate liquidity connectedness among our sample cryptocurrencies, with Bitcoin (BTC) and Litecoin (LTC) playing a significant role in connectedness magnitude. Distinct liquidity clusters for Ethereum (ETH) and Dash are observed for BTC, LTC, and Ripple (XRP). Moreover, liquidity connectedness is more pronounced in the short-run time horizon than in the medium- and long-run time horizons. The time-varying analysis shows that the liquidity connectedness has increased over time, pointing to the potential impact of increasing demand and higher acceptability on individual and institutional investors for this unique asset.

The remainder of the paper unfolds as follows. "Literature review" section provides a summary of the related literature. "Data and methodology" section describes the dataset and methodology. Fourth section presents "Empirical findings". "Conclusion" section concludes the study.

## Literature review

This section provides a brief overview of the literature related to the topic. Cryptocurrencies have demonstrated tremendous potential for integration into the global financial system, albeit their speculative demand has significantly contributed to their volatility and price bubbles (Cheung et al. 2015; Fry and Cheah 2016; Corbet et al. 2018; Bouri et al. 2019b). Cryptocurrencies have also shown hedging/safe-haven potential against traditional assets (Naeem et al. 2020a; Bouri et al. 2020). Additionally, cryptocurrencies carry unique and attractive features for investors, including anonymity, decentralization, little oversight, and low transaction costs. Well-reputed financial institutions have been participating in the cryptocurrency market since 2018. For instance, Fidelity, ICE, and NASDAQ have taken steps to strengthen the cryptocurrency trading infrastructure. In February 2019, the public pensions’ entry into the cryptocurrency market boosted other institutional investors’ confidence (Brauneis et al. 2020).

Consequently, research into cryptocurrency markets has grown exponentially in recent years. Broadly, two strands of literature are related to the topic. The first strand encircles the studies focusing on the liquidity in the cryptocurrency market. The second one summarizes the literature that focused on the connectedness or spillovers among cryptocurrency markets.

The first and emerging strand of literature that looks at liquidity in the cryptocurrency market has not emphasized the connectedness of liquidity among these markets. Kim (2017) and Dyhrberg et al. (2018) suggested that BTC’s attractiveness for retail trading lies in its lower transaction costs. Loi (2018) also regarded various exchanges to compare liquidity. By implementing different low-frequency liquidity indicators, the author found that BTC’s liquidity is typically lower than stocks and that liquidity differs throughout exchanges. Similarly, Smales (2019) suggested that the liquidity for BTC is lower than other safe-haven investments, such as gold. Considering different sets of cryptocurrencies, Brauneis and Mestel (2020) and Wei (2018) indicated a positive (negative) relationship between liquidity and price efficiency (volatility). Koutmos (2018) developed a proxy for liquidity uncertainty by relating it to the market features and trading activity of BTC. Scharnowski (2021) found that BTC’s trading volume correlates with the number of tweets and Google search volume. Then, Baur et al. (2019) documented that BTC’s trading volume undergoes daily and weekly calendar anomalies. By closely relating to this strand of literature, we found many empirical studies documenting the linkage between trading volume and returns in the cryptocurrency market, indicating the need for exploring the determinants of trading activity (Bouri et al. 2019c). Many studies capitalized on transaction data for capturing liquidity in the cryptocurrency market while mainly focusing on BTC, such as Loi (2018), Wei (2018), and Brauneis and Mestel (2018). Overall, this literature strand has explored various facets of cryptocurrency liquidity and predominantly investigates the linkage between liquidity and efficiency. However, no study examined the liquidity connectedness in cryptocurrency markets.

The second strand of empirical works investigated the connectedness of either return or volatility in the cryptocurrency market but considered the connectedness of liquidity in these markets. Table 1 provides a summary of all those works.^6^ A key message from these studies is that cryptocurrency markets become increasingly interconnected over time regarding their returns and volatilities.

**Table 1:** Summary of literature

No	References	Empirical model (s)	Data period	Variables used	Key findings
1	Omane-Adjepong and Alagidede (2019)	Multiscale wavelet method; Linear and nonlinear causality; GJR-GARCH	8 May 2014 to 12 February 2018	BitShare, Bitcoin, Litecoin, Ripple, Monero, Stellar, and DASH	Pairwise ranking for diversification and multiple correlations exist; returns (volatility) interactions are scale- and proxy-sensitive; relatively efficient diversification over the short- and medium-terms; and the direction of shock transmission seems non-homogeneous
2	Balli et al. (2020)	Baruník and Křehlík (2018); Continuous Wavelet Transform; Rolling-Window Wavelet Correlation	5 August 2014 to 23 July 2018	Bitcoin, Litecoin, Ripple, Monero, Stellar, Dash, EPU Index, VIX, OVX, and GVZ	Despite drift resemblance across all phases, the short-term connectedness is considerably higher than the medium- and long-term counterparts; increasing connectedness coincides with the popularity of cryptocurrencies; rising economic uncertainty leads to decreasing connectedness
3	Zięba et al. (2019)	Minimum-Spanning Tree (MST); VAR	01 September 2015 to 02 May 2018	78 cryptocurrencies including Bitcoin	Bitcoin was the essential cryptocurrency before 2017, after which Dogecoin has assumed this leading role; causality exists among cryptocurrencies, apart from Bitcoin
4	Yi et al. (2018)	Diebold and Yılmaz (2014); Diebold and Yılmaz (2014) with LASSO-VAR	4 August 2013 to 1 April 2018; 1 December 2016 to 1 April 2018	52 cryptocurrencies	Market capitalization partly drives the cryptocurrency connectedness; unpopular cryptocurrencies, such as Maidsafe Coin become volatility transmitters
5	Katsiampa et al. (2019)	BEKK-MGARCH	7 August 2015 to 10 July 2018	Bitcoin, Ethereum, and Litecoin	Shock transmission between Litecoin (Ethereum) and Bitcoin is bi-directional; conditional correlations are time-varying and predominantly positive
6	Xu et al. (2020)	TENET Framework	18 April 2016 to 16 May 2019	23 Cryptocurrencies, VIX, Gold Bullion Price, the S&P500 composite index, and the S&P400 commodity chemicals index	Risk spillover is significant; a steady rise in the overall connectedness among cryptocurrencies over time; Bitcoin (Ethereum) is the largest receiver (transmitter) of systemic risk
7	Borri and Shakhnov (2019)	Panel Regression	3 January 2017 to 27 April 2017	Bitcoin price listed at several exchanges	Domestic regulatory changes bring about significant spillovers among cryptocurrencies; relative Bitcoin prices and trading volume rise in countries sharing borders
8	Moratis (2020)	Bayesian Vector Autoregressive Mode	October 2016 to May 2020,	30 largest-cap cryptocurrencies	Spillovers among cryptocurrencies are not solely determined by size; increased spillovers combine with greater market integration; internal factors are more critical than external ones
9	Luu Duc Huynh (2019)	VAR-SVAR Granger Causality; Student' s-t Copulas	8 September 2015 to 4 January 2019	Bitcoin, Litecoin Ethereum, Stellar, and XRP	Ethereum exhibits the potential to decouple from other cryptocurrencies, whereas Bitcoin seems to be a spillover recipient
10	Baumöhl (2019)	Detrended Moving-Average Cross-Correlation; Quantile Cross-Spectral Approach (Baruník and Kley 2019)	1 September 2015 to 29 December 2017	Bitcoin, Litecoin, Ethereum, Stellar Lumens, Ripple, and NEM; Japanese Yen, Euro, Swiss Franc, British Pound, Chinese Yuan, and Canadian Dollar	Cryptocurrencies are not as tightly interconnected as they appear; intra-group (inter-group) interactions under extreme lower quantiles are positive (negative)
11	Ji et al. (2019)	Diebold and Yilmaz (2012, 2015)	7 August 2015 to 22 February 2018	Bitcoin, Litecoin, Ethereum, Stellar, Ripple, and Dash	Return connectedness network is centered around Bitcoin (Litecoin); negative returns are more tightly connected than positive ones; global financial uncertainty effects and trading volume drive spillovers
12	Antonakakis et al. (2019)	TVP-FAVAR Connectedness Framework; DCC-GARCH t-Copula; Dynamic Optimal Portfolio Weights; Dynamic Hedge Ratios; Hedge Effectiveness	7 August 2015 to 31 May 2018	Bitcoin, Bitshares, Ethereum, Ripple, Litecoin, Dash, Monero, Nem, and Stellar	Overall, cryptocurrency connectedness shows huge dynamic changes; amplified prospects for heightened connectedness over time; the magnitude of connectedness is associated with cryptocurrency uncertainty; Ethereum transfer shocks to Bitcoin after the recent hyper-volatility episode of Bitcoin
13	Bouri et al. (2019a)	Time–Frequency Granger-causality Test (Bodart and Candelon 2009)	8 August 2015 to 18 February 2019	Bitcoin, Ethereum, Litecoin, Monero, Ripple, Dash, Stellar, and Nem	In some cryptocurrencies, short- and long-run causalities differ from each other; permanent (transitory) shocks dominate over shorter (longer) horizons
14	Bouri et al. (2019b)	GSADF Test (Phillips et al. 2015); Logistic Regression	7 August 2015 to 31 December 2017	Bitcoin, Litecoin, Ripple, Ethereum, Nem, Stellar, and Dash	Multiple explosivity periods are found in all cases, while explosivity transfers across cryptocurrencies; co-explosivity does not necessarily transfer from bigger to smaller cryptocurrencies
15	Bouri et al. (2019c)	Semi-Parametric Approach (Laurent et al. 2016); Co-Jumping Method (Ma et al. 2019); Logistic Regression	8 August 2015 to 28 February 2019	Bitcoin, Bytecoin, Bitshares, Dash, Dogecoin, Digibyte, Litecoin, Ethereum, Nem, Monero, Stellar, and Ripple	While all cryptocurrencies undergo jumps, some experience co-jumping coinciding with the jumping of the trading volume. This confirms the trading volume's importance for cryptocurrency volatility
16	Fousekis and Tzaferi (2021)	Diebold and Yilmaz (2012); Baruník and Křehlík (2018)	January 2018 to March 2020	Bitcoin, Litecoin, Ethereum, and Ripple	Volume data improves the profitability of technical trading. Rational but uninformed traders can benefit from trend analysis. Positive returns may lead to changes in investor expectations
17	Bouri et al. (2021b)	Diebold and Yilmaz (2012) based on quantile VAR	8 August 2015 to 31 December 2020	Bitcoin, Ethereum, Litecoin, Dash, Monero, Ripple, and Stellar	Connectedness becomes stronger with the magnitude of positive and negative shocks. Return connectedness over extreme market conditions is asymmetric
18	Luu Duc Huynh (2019)	SVAR; Granger causality; Student’s-t Copulas	8 September 2015 to 4 January 2019	Bitcoin, Litecoin, Ethereum, Xrp, and Stellar	Ethereum is disentangled from the spillover network, whereas Bitcoin is the spillover recipient
19	Caporale et al. (2021)	Trivariate GARCH-BEKK	12 August 2015 to 15 January 2020	Bitcoin, Ethereum, and Litecoin	Cyber-attacks influence the spillover transmission between cryptocurrency return and volatility, strengthening the connection and thus reducing opportunities for portfolio diversification
20	Huynh et al. (2020)	*Transfer Entropy*	April 2013 to April 2019	14 Cryptocurrencies	Cryptocurrencies with smaller market capitalization turn out to be shock transmitters than the larger ones

VAR, Vector Auto-Regression; GARCH, Generalized Autoregressive Conditional Heteroskedasticity; MGARCH, Multivariate Generalized Autoregressive Conditional Heteroskedasticity; DCC, Dynamic Conditional Correlation; SVAR, Structural Vector Auto-Regression; TVP-FAVAR, Time-Varying Parameter Factor Augmented VAR; BEKK, Baba, Engle, Kraft, and Kroner; GJR, Glosten-Jagannathan-Runkle; TENET, Tail-Event driven NETwork; GSADF, Generalized Supremum Augmented Dickey-Fuller; LASSO, Least Absolute Selection and Shrinkage Operator

The extent and composition of cryptocurrency interconnectedness exhibit a dynamic behavior. Our work contributes to this literature by joining both strands of literature. We argue that, given the presence of interlinkages in cryptocurrency markets, the interconnectedness of liquidity markets and the dynamics of this liquidity connectedness over time and frequency have a substantial potential for exploration. Most previous studies about cryptocurrency connectedness emphasized the spillover dynamics of return (Xu et al. 2021) or volatility (Bouri et al. 2021a). Studies considering cryptocurrency liquidity examined other dimensions of liquidity, such as BTC’s transaction costs and trading (Kim 2017; Dyhrberg et al. 2018). However, no efforts have been made to investigate liquidity connectedness in the cryptocurrency market. We contribute to this dimension in the following ways. First, this study examines the connectedness or spillovers among cryptocurrency markets’ returns or volatilities. We provide a novel evidence on the interconnectedness of crypto liquidity instead of returns or volatility spillover literature provided in Table 1. To the best of our knowledge, this study is the first to uncover the dynamics of liquidity connectedness in cryptocurrency markets. Thus, second, we contribute to a broad strand of literature by focusing on the connectedness or spillovers among financial markets, such as stock market (Diebold and Yilmaz 2009; Shahzad et al. 2018), bond markets (Christiansen 2007; Ahmad et al. 2018), commodity markets (Diebold et al. 2017; Balli et al. 2019), forex markets (Baruník et al. 2017), and small and medium enterprises (Kou et al. 2014, 2021; Zha et al. 2020). Third, this study explores liquidity connectedness in financial markets, such as the stock market (Chuliá et al. 2020; Inekwe 2020). Finally, this study takes the literature on cryptocurrency liquidity one step further by exploring the liquidity linkages among cryptocurrencies, thereby adding to the previous works on crypto liquidity or its linkage with price efficiency (Kim 2017; Dyhrberg et al. 2018; Loi 2018; Smales 2019; Wei 2018; Brauneis and Mestel 2018; Koutmos 2018; Baur et al. 2019; Bouri et al. 2019d).

## Data and methodology

### Data

We select the six most liquid cryptocurrencies, namely, BTC, LTC, ETH, XRP, Monero (XMR), and Dash. At the moment, these cryptocurrencies are the most important in terms of trading volume. Within our sample, as of 2019, in terms of trading volume and market capitalization, BTC is the largest cryptocurrency, followed by ETH, LTC, XMR, Dash, and XRP (Al-Yahyaee et al. 2020). We consider these six cryptocurrencies as they attract considerable attention from investors, policymakers, and academics, such as Al-Yahyaee et al. (2020). The liquidity and popularity of these cryptocurrencies are critical elements of our choice to include them in this study. All cryptocurrencies’ daily prices and trading volumes constitute our dataset, spanning from August 7, 2015, to December 28, 2019. The data were gathered from coinmarketcap.com, which is the most popular data hub for cryptocurrency information and has been extensively used by many recent works (Yi et al. 2018; Omane-Adjepong and Alagidede 2019).

### Methodology

Our methodology consists of three parts. The first part introduces the two measures that we use to compute liquidity in the cryptocurrency market. The second one lays out the details of the connectedness framework proposed by Diebold and Yilmaz (2012). Finally, the third part is the methodology section, that is, the frequency connectedness framework of Brunik and Krehlik (2016). This framework allows us to capture the time–frequency dynamics of the liquidity connectedness among cryptocurrency markets.

#### Liquidity measures

In this study, we use two liquidity measures. The first one is the measure of Amihud (2002) given in Eq. (1), which was used by several studies (Kamara et al. 2008; Korajczyk and Sadka 2008; Marshall et al. 2012). Such studies highlighted its superiority over other low-frequency liquidity proxies, which often do a poor job in capturing liquidity in financial markets. Brauneis and Mestel (2018) also used this measure for the computing liquidity for the cryptocurrency market.

1 $${\text{LIQ}}_{{\text{t}}} = \frac{{\left| {{\text{Ret}}_{{\text{t}}} } \right|}}{{{\text{Vol}}_{{\text{t}}} }},$$

where ${\text{Ret}}_{{\text{t}}}$ and ${\text{Vol}}_{{\text{t}}}$ are the returns and dollar volumes on day *t* for a given cryptocurrency, respectively.

The second liquidity measure used in this study is the volatility-over-volume ($VoV_{t}$). Introduced by Fong et al. (2018), this measure minimizes the impact of extreme values using a range of high ($H_{t}$) and low ($L_{t}$) prices, instead of absolute returns, and the square root of the volume on a given day. This measure is given as follows:

2 $${\text{VoV}}_{{\text{t}}} = \frac{{{\text{ln}}\left( {\frac{{{\text{H}}_{{\text{t}}} }}{{{\text{L}}_{{\text{t}}} }}} \right)}}{{\sqrt {{\text{Vol}}_{{\text{t}}} } }}.$$

#### Liquidity connectedness of Diebold and Yilmaz (2012)

We first apply the spillover model of Diebold and Yilmaz (2012) to compute the liquidity connectedness across our sample cryptocurrencies. Moreover, we implement the connectedness framework of Baruník and Křehlík (2018) to further explore the time–frequency domain aspect of liquidity connectedness.

#### Liquidity connectedness of Diebold and Yilmaz (2012)

Following Diebold and Yilmaz (2012), we build on an N-variable vector autoregression (VAR) fitted to the cryptocurrency illiquidity series. To begin, we consider an N-dimensional vector $y_{t} = { }\left( {y_{1t} , \ldots ,u = y_{nt} } \right)^{^{\prime}}$ holding the illiquidity series of N cryptocurrencies, which is modeled by a covariance stationary VAR (p) as $y_{t} = \sum\nolimits_{i = 1}^{p} {\phi_{i} } y_{t - i} + \varepsilon_{t}$. In this equation, $\varepsilon_{t} { }\sim { }N\left( {0,\Sigma_{\varepsilon } } \right)$ is a vector of independently and identically distributed disturbances and $\phi_{i}$, for $i = 1, \ldots ,{\text{p}}$ coefficient matrices. Consequently, a moving average (MA) depiction driven from the VAR model, therefore, results in an MA ($\infty$) process, $y_{t} = \sum\nolimits_{i = 0}^{\infty } {\psi_{i} } \varepsilon_{t - i}$, where $\psi_{i}$ is a coefficient matrix of order $N \times N$, which is recursively computed through $\psi_{i} = \phi_{1} \psi_{i - 1} + \phi_{2} \psi_{i - 2} + \cdots + \phi_{p} \psi_{i - p}$, where $\psi_{0}$ is the identity matrix.

Subsequently, we follow Koop et al. (1996) and Pesaran and Shin (1998) to achieve orthogonality through the generalized framework. Hence, a given series $j$’s contribution to another series $i$’s *H*-step-ahead generalized forecast error variance is represented by $\xi_{ij} \left( {\text{H}} \right)$, which is estimated as follows:

3 $$\xi_{ij} \left( {\text{H}} \right) = \frac{{\sigma_{jj}^{ - 1} \mathop \sum \nolimits_{h = 0}^{H - 1} \left( {\Im_{i}^{\prime } \psi_{h} \sum \Im_{j} } \right)^{2} }}{{\mathop \sum \nolimits_{h = 0}^{H - 1} \left( {\Im_{i}^{\prime } \psi_{h} \sum \psi_{h}^{\prime } \Im_{i} } \right)^{2} }},$$

where $\sum$ and $\sigma_{jj}$ represent the covariance matrix of errors and the *j*th component of the standard deviation’s diagonal, respectively. For an *i*th component, $\Im_{i}$ takes a value of 1, and 0 if otherwise.^7^ In the non-orthogonalized Vector Autoregressives (VAR’s) infinite Moving Average (MA) representation, $\psi_{h}$ represents a coefficient matrix with the multiplication of *h*-lagged errors.

Accordingly, the pairwise connectedness from series *j* to series *i* is given as follows:

4 $${\Omega }_{i \leftarrow j}^{H} = \xi_{ij} \left( {\text{H}} \right).$$

Consequently, we can capture the total directional connectedness to (from) other series to series *j (i)*. Diebold and Yilmaz (2012) noted that the total directional connectedness can be obtained by dividing the off-diagonal sum of columns (rows) by the sum of all elements, which is represented as follows:

5 $${\Omega }_{i \leftarrow \bullet }^{H} = \frac{1}{N}\mathop \sum \limits_{{\begin{array}{*{20}c} {j = 1} \\ {j \ne i} \\ \end{array} }}^{N} \xi_{ij} \left( {\text{H}} \right),$$

6 $${\Omega }_{ \bullet \leftarrow j}^{H} = \frac{1}{N}\mathop \sum \limits_{{\begin{array}{*{20}c} {i = 1} \\ {i \ne j} \\ \end{array} }}^{N} \xi_{ij} \left( {\text{H}} \right).$$

Similarly, we can obtain the total or system-wide connectedness. According to Diebold and Yilmaz (2012), this connectedness can be computed by dividing the sum of other (from others) elements by the sum of all its elements:

7 $${\Omega }^{H} = \frac{1}{N}\mathop \sum \limits_{{\begin{array}{*{20}c} {i,j = 1} \\ {i \ne j} \\ \end{array} }}^{N} \xi_{ij} \left( {\text{H}} \right).$$

#### Frequency connectedness of Baruník and Křehlík (2018)

Next, we estimate the cross-market connectedness over short- and long-term horizons. Accordingly, we resort to Baruník and Křehlík (2018) frequency connectedness model. The model decomposes the variance into spectral components and computes the connectedness over short- and long-term horizons.

Accordingly, the Fourier transform of the coefficients $\Gamma_{k}$, for $i = \sqrt { - 1}$, helps us ascertain the frequency response function, $\Gamma \left( {\Im^{ - i\theta k} } \right) = \mathop \sum \limits_{k} \Im^{ - i\psi k} \Gamma_{k}$. At a given frequency band, θ, the Fourier Transform for ${\text{MA}}(\infty)$ processes defines XY’s spectral density and is given as follows:8where $T_{XY} \left(\uptheta \right)$ is the power spectrum that maps the distribution of $XY_{t}$’s variance for each $\psi$. Frequency domains can also be alternatively described through covariance’s spectral decomposition, which is expressed as $Exp\left( {XY_{t}, XY^{\prime }_{t - k} } \right) = \mathop \smallint \limits_{ - \varphi }^{\varphi } T_{\phi } \left(\uptheta \right)\Im^{{i{\text{f}}k}} d\uptheta$.

Following Baruník and Křehlík (2018), the cross-spectral density of the interval, $l = \left( {c,d} \right):c,d \in \left( { - \varphi ,\varphi } \right),c < d,$ is estimated and given as follows:

9 $$\mathop \sum \limits_{\uptheta } \hat{\Gamma }\left( f \right)\widehat{\sum }\hat{\Gamma }^{\prime } \left(\uptheta \right),$$

for $$\uptheta \in \left\{ {{\raise0.7ex\hbox{${cK}$} \!\mathord{\left/ {\vphantom {{cK} {2\pi }}}\right.\kern-\nulldelimiterspace} \!\lower0.7ex\hbox{${2\pi }$}}, \ldots ,{\raise0.7ex\hbox{${dK}$} \!\mathord{\left/ {\vphantom {{dK} {2\pi }}}\right.\kern-\nulldelimiterspace} \!\lower0.7ex\hbox{${2\pi }$}}} \right\}$$, where

10 $$\hat{\Gamma }\left(\uptheta \right) = \mathop \sum \limits_{k = 0}^{K - 1} \hat{\Gamma }_{k} \Im^{{ - 2i\varphi\uptheta {/}K}}$$

and

$$\widehat{\sum } = {\raise0.7ex\hbox{${\hat{\varepsilon }^{\prime } \hat{\varepsilon }}$} \!\mathord{\left/ {\vphantom {{\hat{\varepsilon }^{\prime } \hat{\varepsilon }} {\left( {{\Omega } - y} \right)}}}\right.\kern-\nulldelimiterspace} \!\lower0.7ex\hbox{${\left( {{\Omega } - y} \right)}$}},$$

where $y$ represents adjustments that correspond to the loss of degrees of freedom, which strictly depends on the VAR framework.

Consequently, a frequency-based decomposition of the impulse response is given by $\hat{\Gamma }\left( l \right) = \mathop \sum \limits_{f} \hat{\Gamma }\left(\uptheta \right)$, where the generalized FEVDs are calculated as follows:

11 $$\left( {\hat{\Re }_{l} } \right)_{j,m} = \mathop \sum \limits_{f} \hat{\rho }_{j} \left(\uptheta \right)\left( {\hat{P}\left(\uptheta \right)} \right)_{j,m} ,$$

where $\left( {\hat{P}(\uptheta )} \right)_{j,m} = {{\hat{\delta }_{ll}^{ - 1} \left( {\left( {\hat{\eta }(\uptheta )\widehat{\sum }} \right)_{j,m} } \right)^{2} } \mathord{\left/ {\vphantom {{\hat{\delta }_{ll}^{ - 1} \left( {\left( {\hat{\eta }(\uptheta )\widehat{\sum }} \right)_{j,m} } \right)^{2} } {\left( {\hat{\Gamma }\left(\uptheta \right)\widehat{\sum }\Gamma^{\prime } (\uptheta )} \right)_{j,j} }}} \right. \kern-\nulldelimiterspace} {\left( {\hat{\Gamma }\left(\uptheta \right)\widehat{\sum }\Gamma^{\prime } (\uptheta )} \right)_{j,j} }}$ and $\hat{\Re }_{j} (\uptheta ) = {{\left( {\hat{\Gamma }(\uptheta )\widehat{\sum }\Gamma^{\prime } (\uptheta )} \right)_{j,j} } \mathord{\left/ {\vphantom {{\left( {\hat{\Gamma }(\uptheta )\widehat{\sum }\Gamma^{\prime } (\uptheta )} \right)_{j,j} } {(f)_{j,j} }}} \right. \kern-\nulldelimiterspace} {(f)_{j,j} }}$ denote the estimates for the generalized causation spectrum and the weighted fraction, respectively; $f$ can be computed from $\mathop \sum \limits_{f} \hat{\Gamma }(\uptheta )\widehat{\sum }\hat{\Gamma }^{\prime } (\uptheta )$. Finally, the frequency connectedness estimates are achieved by substituting $\left( {\hat{\Re }_{k} } \right)_{j,m}$ into the abovementioned connectedness matrices.

## Empirical findings

### Liquidity connectedness and clustering

**Fig. 1:**
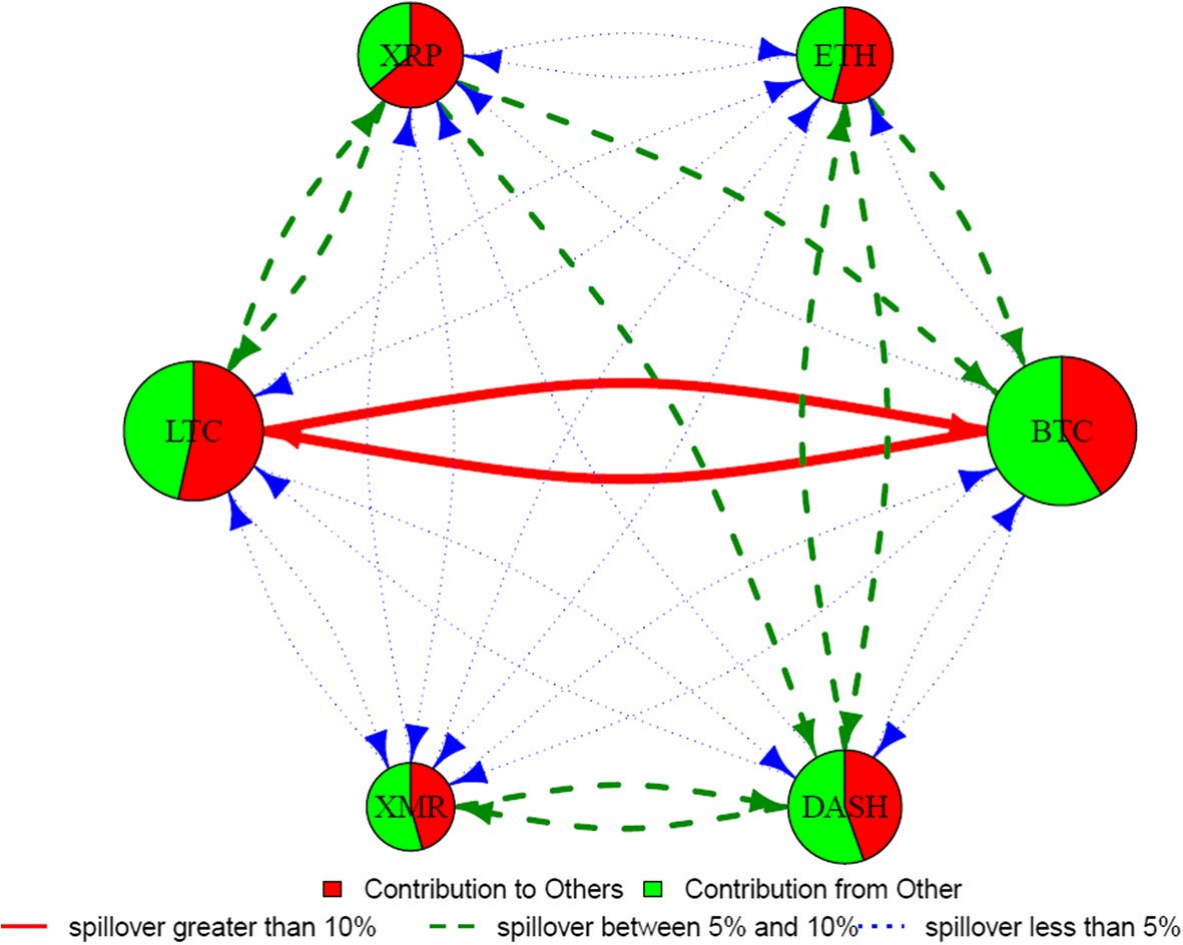
Spillover diagram using DY approach. *Note*: This network graph illustrates the degree of total connectedness in a system that consists of the six cryptocurrencies over the full sample period. Total connectedness is measured using the Diebold-Yilmaz framework. The size of the node shows the magnitude of contribution of each variable to system connectedness, while the color indicates the origin of connectedness. In particular, the red color implies contribution from the variable under consideration to the other variables of the system and the green color means contribution from the other variables to the variable under analysis. The color and shape of the arrows refer to the strength of connectedness. The red colour and full line arrows represent strong spillovers while green and blue colour arrows show medium and weak liquidity spillovers, respectively

We use the spillover approach of Diebold and Yilmaz (2012) to estimate the liquidity connectedness among six major^8^ cryptocurrencies, namely, BTC, LTC, ETH, XRP, XMR, and Dash. Figure 1 depicts a liquidity connectedness network that shows the direction, magnitude, and strength of liquidity spillovers from each currency to all other coins and backward. In terms of size, BTC and LTC are the most significant contributors in the liquidity connectedness network, followed by Dash and XRP, whereas ETH and XMR are the smallest contributors. Moreover, in terms of net spillovers, BTC, Dash, and XMR are net receivers of liquidity spillovers, whereas LTC, XRP, and ETH are net transmitters.

BTC is the leading net receiver of liquidity spillovers. BTC receives strong liquidity spillovers from LTC and medium spillovers from XRP and ETH, whereas the rest of the currencies transmit weak liquidity spillovers to BTC. Conversely, BTC transmits strong liquidity spillovers to LTC and weak spillovers to all other currencies. Interestingly, despite being the most liquid (Wei 2018) and the largest cryptocurrency, BTC receives substantial liquidity spillovers from smaller currencies, that is, XRP. This observation questions the dominance of BTC in the cryptocurrency market concerning return (Antonakakis et al. 2019; Ji et al. 2019) and volatility (Yi et al. 2018) spillover transmitter. However, owing to the concept of liquidity commonality, we suggest that an increase in the overall cryptocurrency market liquidity will enhance BTC’s liquidity more than any other currency because it reigns as a well-established and most significant cryptocurrency.

Similar to BTC, Dash and XMR are also net receivers of liquidity spillovers. Dash receives medium liquidity spillovers from ETH and XRP and transmits the same to XMR and ETH. From another perspective, XMR receives medium spillovers from Dash but weak spillovers from the rest of the currencies and only transmits medium liquidity spillovers to Dash. Interestingly, the smallest cryptocurrency in our sample, that is, Dash, receives substantial liquidity spillover from ETH and XRP, which are relatively smaller cryptocurrencies than BTC.

Unlike BTC, its fork, LTC, is a net transmitter of liquidity spillovers, which transmits strong (medium) liquidity spillovers to BTC (XRP), whereas other currencies receive weak liquidity spillovers from LTC. Similarly, ETH and XRP are also net transmitters of liquidity spillovers. In addition to transmitting medium liquidity spillovers to BTC and Dash, ETH transmits weak liquidity spillovers to other currencies. Remarkably, XRP is the most connected cryptocurrency in terms of spillovers transmission strength, which transmits substantial liquidity spillovers to more currencies than any other net transmitters. XRP’s liquidity transmission strength could be attributed to its unique payment system, fast transaction process, and lower transaction cost.^9^

Following the network-based liquidity connectedness analysis, we move to estimate and analyze liquidity clustering in the cryptocurrency market. Ji et al. (2019) reported that cryptocurrencies tend to show similar (different) returns and volatility patterns, which potentially make them compliments (diversifiers). Additionally, using different Eurozone credit market sectors, Shahzad et al. (2019) maintained that identifying different risk clusters can help devise diversification strategies. Thus, using liquidity clustering analysis, we posit that currencies appearing in distinct liquidity clusters could serve diverse investment objectives, that is, short-/long-term, and serve as complements or alternatives.

**Fig. 2:**
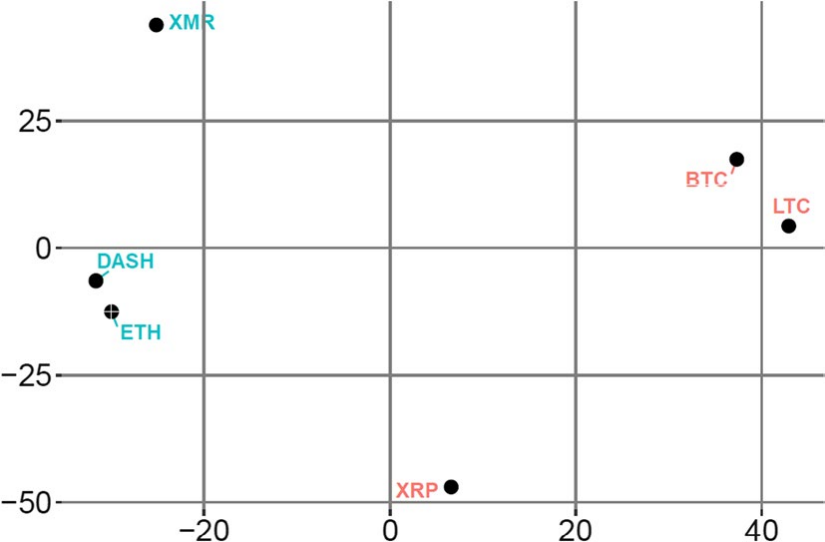
Cluster based on decomposed DY approach. *Note*: This figure shows the symmetric part of the connectedness table for the full sample period. Two colours (red and blue) show two distinct risk clusters formed through hierarchical clustering

We use the symmetric part of the connectedness table to form liquidity clusters. Figure 2 shows the liquidity clusters, where red and blue identify two distinct liquidity clusters. The liquidity clustering shows that BTC, LTC, and XRP form a liquidity cluster, whereas Dash, ETH, and XMR form another distinct liquidity cluster. BTC/LTC and Dash/ETH pairs show tight clustering within the clusters, whereas XRP and XMR have dispersed positioning in each cluster. These observations reinforce our liquidity connectedness findings, showing that BTC and LTC are strongly connected. Additionally, these findings confirm moderate liquidity connectedness between ETH and Dash. Conversely, the loose clustering of XMR validates its least connectivity to other currencies.

Overall, the full sample results show moderate liquidity connectedness among cryptocurrencies, with BTC and LTC being the prominent actors in terms of magnitude. They also form a distinct liquidity cluster along with XRP. Consequently, the BTC/LTC pair could serve as a strong complement in a portfolio as they also maintain a strong (weak) return (volatility) connectedness (Ji et al. 2019). Similarly, the LTC/XRP pair appears to present promising complementary attributes because of its medium liquidity connectedness, low volatility connectedness (Yi et al. 2018), and price co-explosivity (Bouri et al. 2019e). In an economic context, the complimentary liquidity attributes of different cryptocurrencies suggest profitable avenues for an investor operating in the cryptocurrency market. Specifically, these opportunities can provide sizable economic profits when the right combination of cryptocurrencies is used to form investment portfolios, that is, BTC and LTC. Moreover, long-term investors holding portfolios of complementary cryptocurrencies can benefit from price explosiveness episodes frequently observed in the cryptocurrency market. Hence, investors can group complementary cryptocurrencies into their portfolios to make most of the opportunities available in the market. Furthermore, the weakest liquidity connectedness and separate clustering of XMR are potential substitute investments among cryptocurrencies. One possible explanation of the distinction for XMR could be its extreme secrecy of transaction processes, which is distinctive from the leading cryptocurrencies, that is, BTC.

### Liquidity connectedness and clustering in frequency domains

**Fig. 3:**
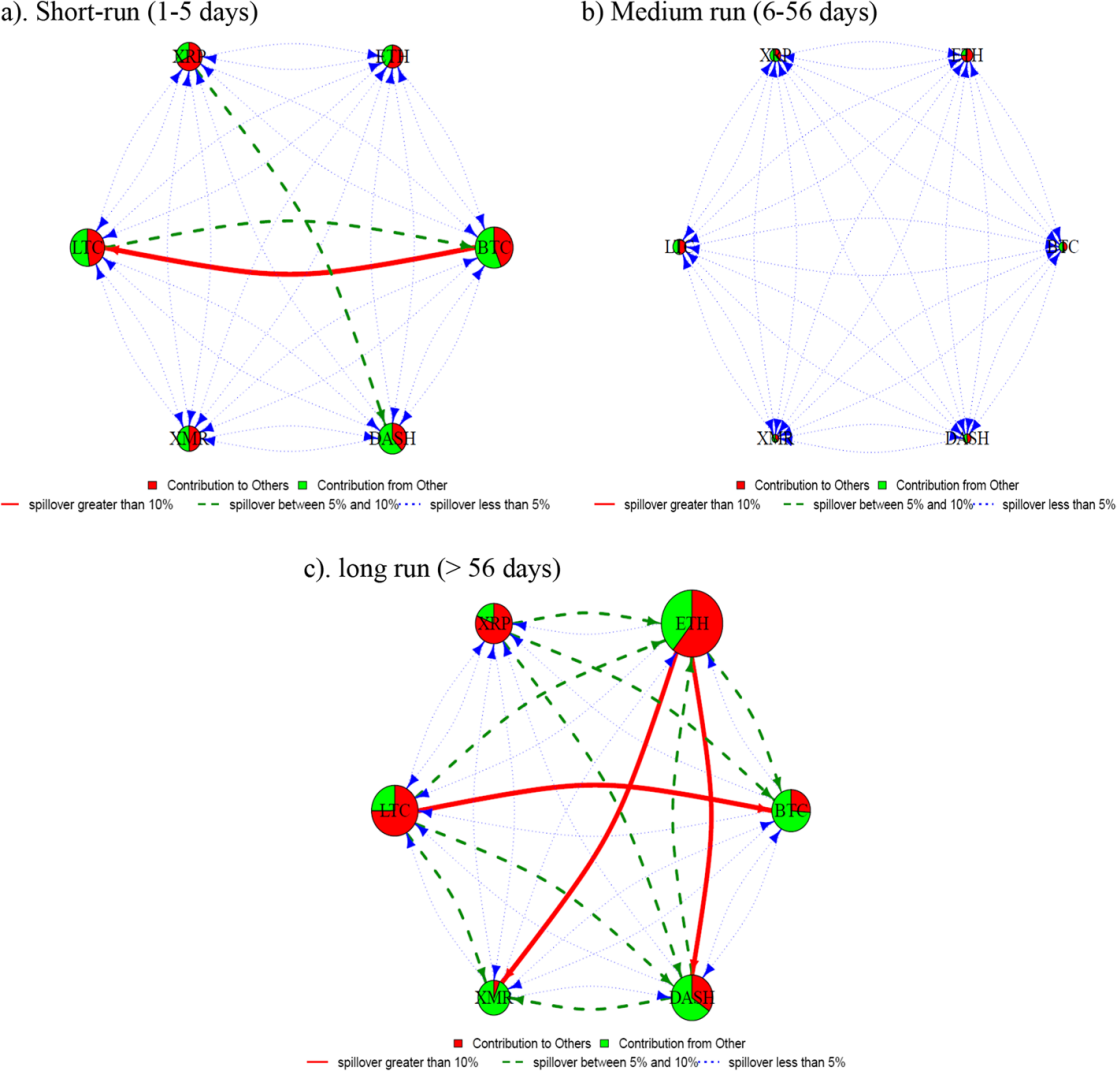
Frequency domain spillover using BK approach

Furthermore, we refer to Baruník and Křehlík (2018) to estimate and analyze the liquidity connectedness among cryptocurrencies in three frequency domains, namely, short run (1–5 days), medium run (6–56 days), and long run (> 56 days). We choose these frequency bands based on the literature (Balli et al. 2020; Baruník and Křehlík 2018; Hasan et al. 2021). Collectively, frequency domain analysis shows moderately strong connectedness in magnitude and strength terms for the long run as opposed to low and negligible connectedness in short- and medium-run frequencies, respectively. Specifically, in the short run (Fig. 3a), we observe moderate connectedness among BTC and LTC, and these currencies also heavily contribute to the connectedness magnitude followed by Dash and XRP. Except for BTC/LTC and Dash/XRP pairs, relatively moderate liquidity connectedness among cryptocurrencies in the short run shows that investors tend to trade the most liquid currencies more frequently than their less liquid counterparts, that is, ETH and XMR. Additionally, factors such as strong return connectedness (Ji et al. 2019; Naeem et al. 2021c) and frequent co-price explosivity (Bouri et al. 2019c) of BTC/LTC and LTC/XRP pairs may also play a role in liquidity connectedness for these currencies in the short run. Furthermore, Fig. 3b presents the medium-run liquidity connectedness network. Notably, the magnitude and strength of connectedness are weaker in the medium run than in other frequency domains. For example, we observe a significant decline in the magnitude and strength of total spillovers from and to BTC in the medium-run analysis. However, all the cryptocurrencies maintain a similar net spillover status in the short and medium terms.

Furthermore, Fig. 3c presents the long-run results of liquidity connectedness, showing several changes in the network. First, we observe a substantial increase in the magnitude of connectedness for ETH as it surpasses BTC and LTC. Second, the strength of incoming and outgoing spillovers for ETH is much stronger than that of any other cryptocurrency. Notably, being the second-largest cryptocurrency after BTC, ETH displays a vital role in liquidity transmission within the cryptocurrency market in the long run. In a different vein, Antonakakis et al. (2019) reported the enhanced importance of ETH in the recent past as it takes the role of the leading net return spillover transmitter surpassing BTC. Finally, although all the cryptocurrencies maintain their net spillover status, the majority of liquidity connectedness, in the long run, is limited to liquidity spillovers to and from ETH. For example, ETH transmits more substantial spillovers to Dash and XMR in the long run, unlike negligible spillovers in the short and medium run. Conversely, BTC receives relatively higher liquidity spillovers and shows minimal spillover transmission potential in the long run than other frequency domains.

**Fig. 4:**
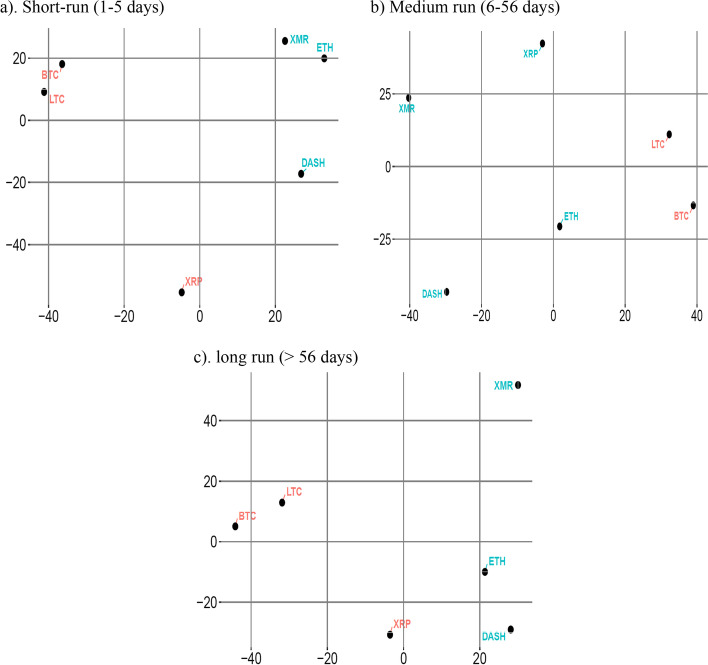
Frequency domain spillover using BK approach

Next, we apply frequency domain analysis on liquidity clustering to analyze liquidity clustering in the cryptocurrency market for the short, medium, and long run. Figure 4a–c presents the liquidity clustering of cryptocurrencies in three frequency domains. We observe similar liquidity clustering for the short and long run, showing that some cryptocurrencies have similar liquidity dynamics in the short and long run. However, the medium-run liquidity clustering shows that XRP forms a cluster with ETH, XMR, and Dash instead of the BTC/LTC pair. Additionally, the clusters in the medium run are more widely spread compared with that in the other frequency domains. These observations coincide with our finding of medium-run connectedness analysis that shows an overall weaker liquidity connectedness among cryptocurrencies in the medium run as compared with the short and long run.

### Dynamic connectedness analysis

The network connectedness approach discussed above provides essential insights into liquidity spillovers among cryptocurrencies over time. However, the network approach overlooks the time-varying aspect of liquidity spillovers. This aspect is very important in cryptocurrencies because the cryptocurrency market has experienced many changes during the sample period under consideration. For example, the price of many cryptocurrencies increased many-folds, the overall cryptocurrency market received enhanced mainstream media coverage, future and derivative contracts launched for cryptocurrencies, and many governments worldwide authorized cryptocurrency trading.

**Fig. 5:**
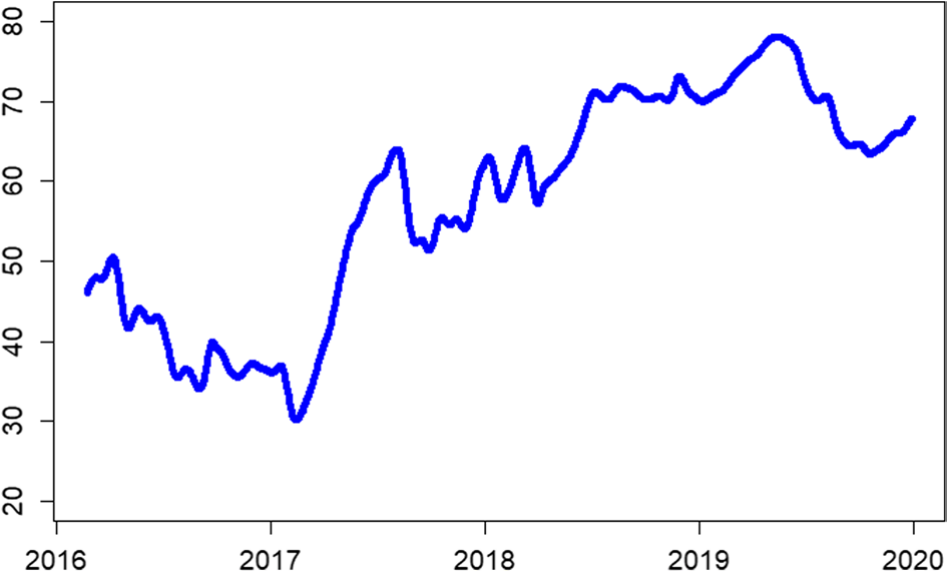
Rolling-window based total return spillover index based on the DY approach. *Note*: 200 days rolling window, lag order 12 based on AIC

Therefore, we estimate the dynamic connectedness spillovers using a 200-day rolling window with a lag order of 12 based on Akaike Information Criteria (AIC) to capture the time-varying liquidity spillovers. The full-sample dynamic connectedness presented in Fig. 5 shows time-varying liquidity spillovers. We observe a declining trend from the start of the sample period to early 2017, when liquidity connectedness increases and peaks in October 2017. The initial decline in liquidity connectedness can be attributed to the hacking of Bitfinex and British exit from the EU. Contrarily, factors such as Japan declaring BTC as a legal tender on April 1, 2017, led to enhanced liquidity connectedness.

In post-2017, the liquidity connectedness shows a gradual upward trend, with the connectedness index approaching 80% in early 2019. Such higher liquidity connectedness in the cryptocurrency market is attributable to developments such as introducing real-time settlement systems and cryptocurrency trading pairs, that is, XRP/BTC. These developments have significantly reduced the settlement time for transactions and enhanced the exchange of value across borders. The liquidity connectedness trend in our sample follows a similar pattern of connectedness as reported by Ji et al. (2019) in the case of return connectedness for major cryptocurrencies.

Notably, in 2017, the prices of sample cryptocurrencies exponentially increased,^10^ and many new currencies entered the digital currency market. Thus, the increased awareness and profitability of major cryptocurrencies, that is, BTC, ETH, and XRP, increasingly attracted investors to this new investment asset, consequently enhancing the liquidity and liquidity connectedness of the entire market. Additionally, Będowska-Sójka et al. (2020) reported a bidirectional causality between volatility and liquidity in the cryptocurrency market. Therefore, volatility attracts investors to the cryptocurrency market and leads to enhanced liquidity. This finding is particularly relevant to the cryptocurrency market as most investors use cryptocurrencies for speculative purposes. Furthermore, the phenomenon of increased liquidity and liquidity connectedness in the cryptocurrency market could be explained by the demand-side explanation of commonality in liquidity. That is, increased demand for an asset by institutional investors^11^ could increase the commonality in liquidity.

### Dynamic connectedness analysis in frequency domains

**Fig. 6:**
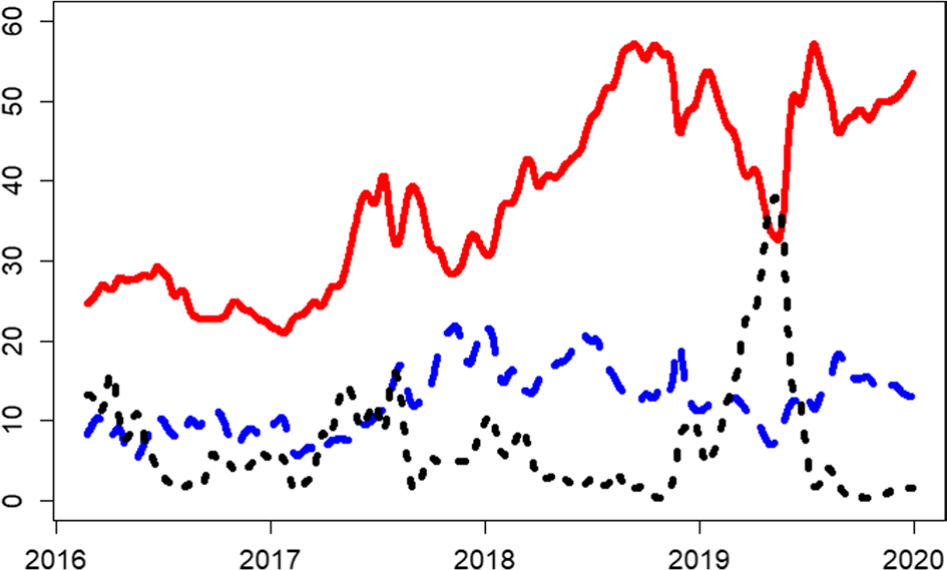
Rolling-window based total return spillover index based on the BK approach. *Note*: 200 days rolling window, lag order 12 based on AIC. Short-run (0–5 days) is in red color; medium-run (6–56 days) is in blue color; long-run (more than 56 days) in black color

We analyze the dynamic connectedness in three frequency domains, that is, short, medium, and long run. Figure 6 shows that liquidity connectedness is higher in the short run than in the medium and long run. Although we observe a similar time-varying pattern for all frequency domains, some exceptions exist. For example, from the start of the sample period to early 2017, all frequency domains show a similar downward trend. Similarly, all frequency domains show an upward trend in the second and third quarters of 2017 and fluctuating behavior after the second quarter in 2018. From another perspective, a distinct contrast appears between short- and long-run connectedness in mid-2019, where short-run connectedness sharply declines, whereas long-run connectedness shows a sharp increase.

In total, the dynamic analysis shows a steady increasing trend in liquidity connectedness for cryptocurrencies. However, a large portion of this connectedness is attributed to short-run connectedness, which indicates the contagion effect. This observation of the contagion effect in the cryptocurrency market has also been reported in the volatility connectedness in cryptocurrency markets (Yi et al. 2018).

### Robustness check

**Fig. 7:**
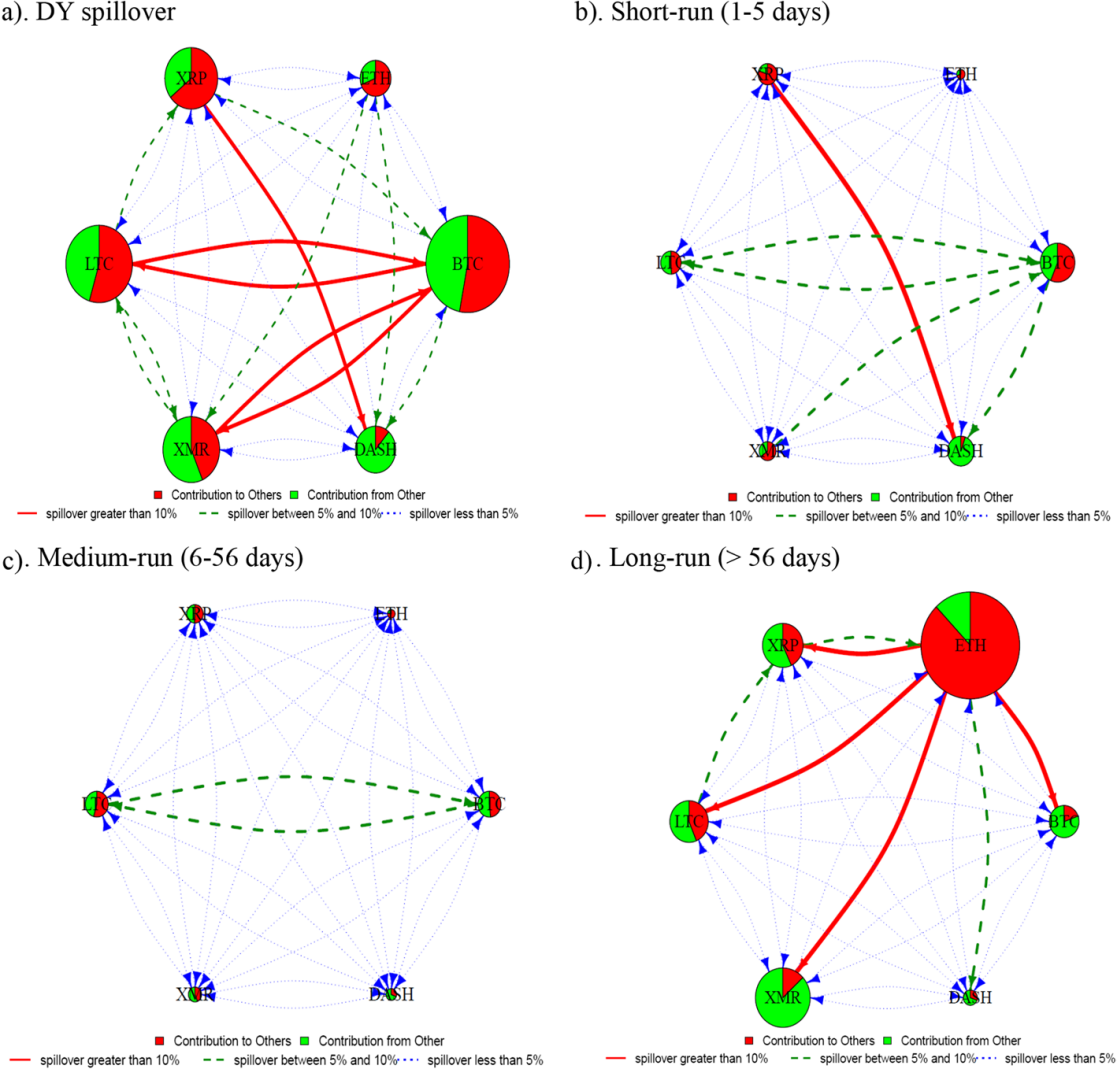
Robustness check using VoV—spillover networks

We use an alternative measure of liquidity developed by Fong et al. (2018) to further validate the findings of our liquidity connectedness and clustering results. Figure 7a–d shows the full-sample and three frequency domains, that is, short-, medium-, and long-run network connectedness results. In terms of connectedness magnitude, we observe similar results for all cryptocurrencies except for Dash and ETH, as the magnitude of these currencies registers a decline in full-sample analysis. Additionally, BTC shows increased strength of connectedness, whereas XRP experiences a fall. We also observe an increased bidirectional connectedness between BTC and XMR. Moreover, the frequency domain connectedness results show a very similar picture, as discussed earlier, using Amihud’s (2002) measure of liquidity.

**Fig. 8:**
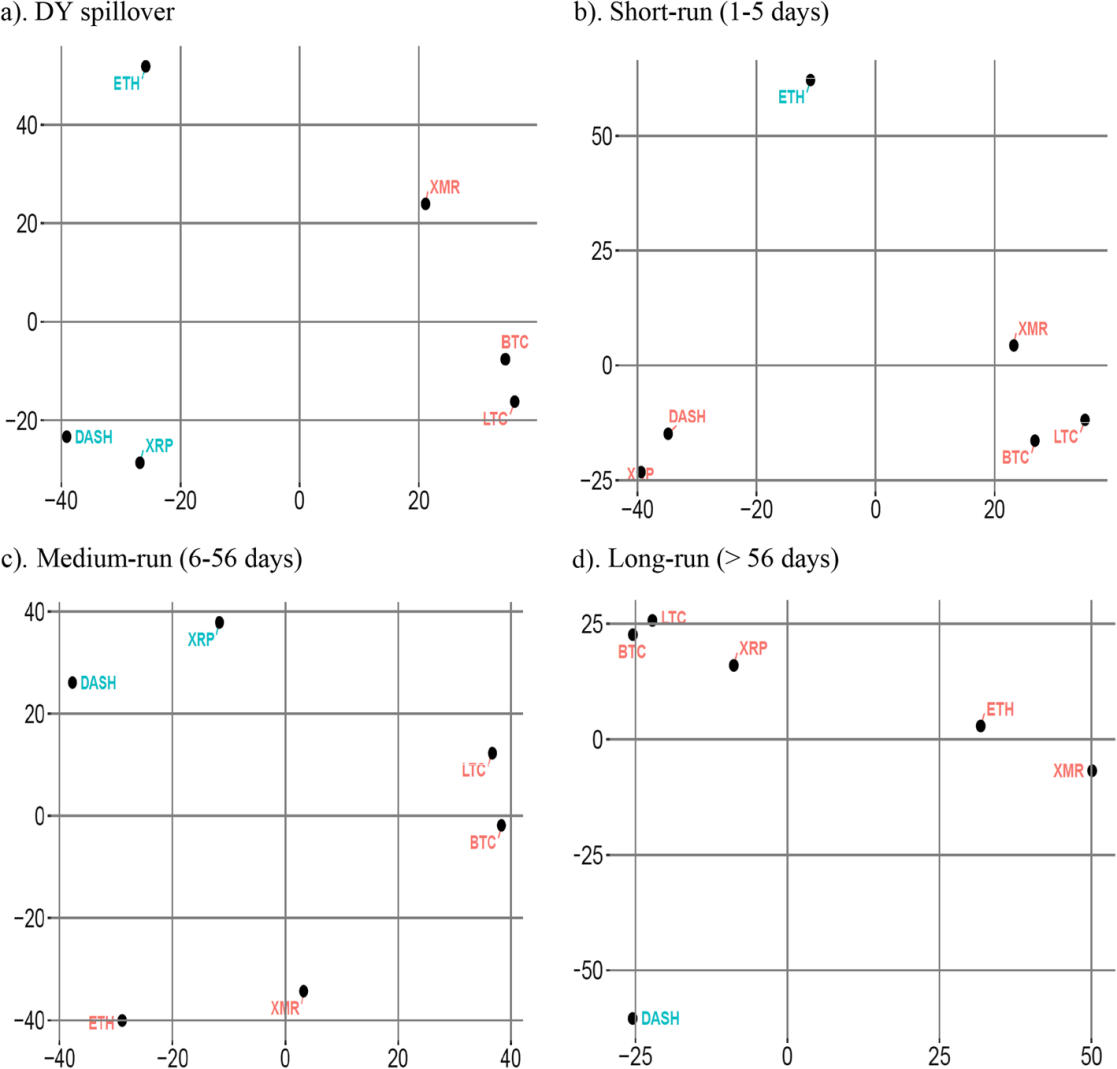
Robustness check using VoV—clusters based on decomposed spillover tables

In the liquidity clustering results presented in Fig. 8a–d, BTC, LTC, and XMR appear in one cluster for the full-sample and frequency domains. These results are somewhat different from our earlier estimates as XMR replaces XRP to form a cluster with BTC and LTC. One potential explanation of the separate clustering of XRP from BTC and LTC is that the return connectedness of XRP with BTC and LTC is more robust than volatility connectedness (Ji et al. 2019). In addition, volatility-over-volume measures use volatility to measure liquidity instead of returns. We observe some changes in the frequency domains of liquidity clustering. However, the BTC/LTC pair always appears in the same cluster, showing the complementary nature of assets under different scenarios. From another perspective, Dash and ETH appear in different liquidity clusters, indicating the substitution opportunities in different frequency domains. Nevertheless, the spread of liquidity clustering in the full-sample and frequency domains remains unaffected.

**Fig. 9:**
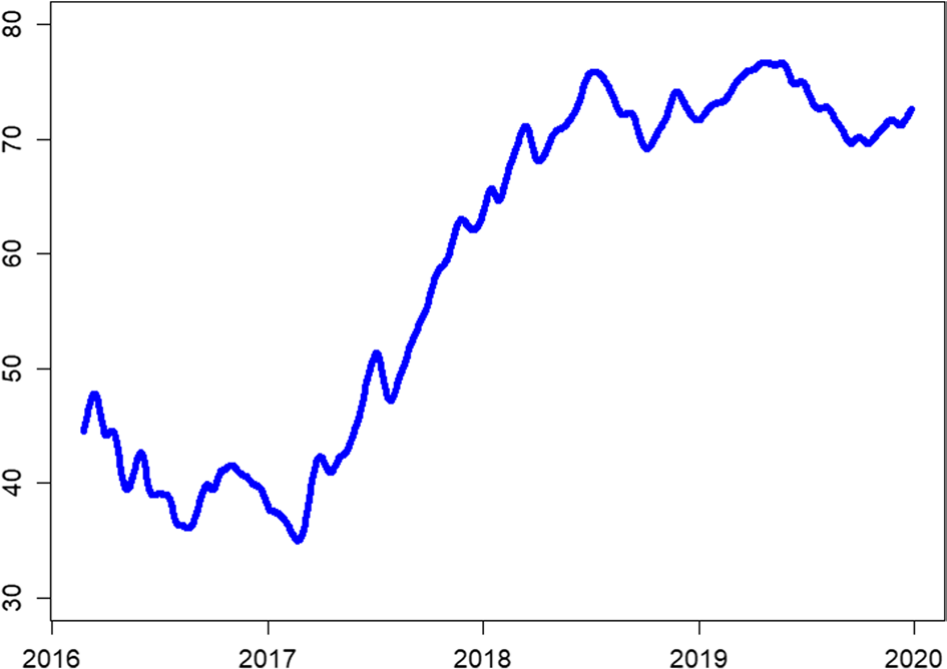
Rolling-window based total return spillover index based on the DY approach. *Note*: 200 days rolling window, lag order 12 based on AIC

**Fig. 10:**
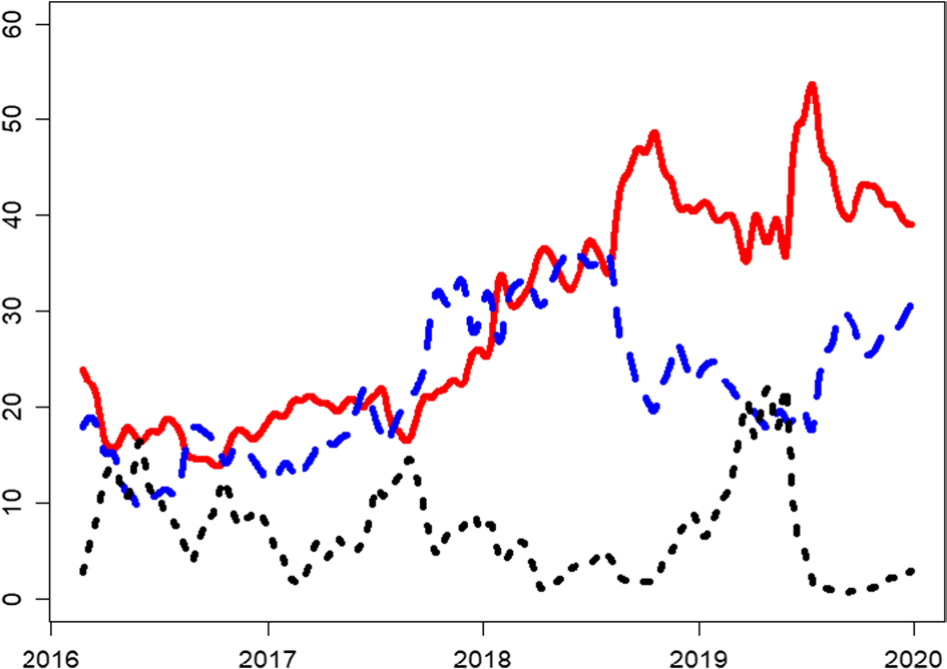
Rolling-window based total return spillover index based on the BK approach. *Note*: 200 days rolling window, lag order 12 based on AIC. Short-run (0–5 days) is in red color; medium-run (6–56 days) is in blue color; long-run (more than 56 days) in black color

Finally, we perform the dynamic connectedness analysis using the volume over volatility liquidity measure in Figs. 9 and 10. For the full sample, the alternate measure captures a similar magnitude and trends. However, we note reduced (heightened) connectedness index levels for short (medium)-run frequency domains, whereas the long-run connectedness remains unchanged.

## Conclusion

This study explores the dynamics of liquidity connectedness in the cryptocurrency market using several static and dynamic connectedness approaches. We use six major cryptocurrencies based on market capitalization and the availability of comprehensive time series data. Using the DY2012 network-based spillover approach, we report a moderate liquidity connectedness among sample cryptocurrencies, with BTC and LTC playing a significant role concerning the magnitude of connectedness. Conversely, XMR and Dash are the least connected currencies in the liquidity network. Additionally, in our liquidity clustering analysis, BTC and LTC, along with XRP, form a distinct liquidity cluster, whereas we observe separating clustering between ETH, XMR, and Dash.

Furthermore, our BK2018 analysis reveals that liquidity connectedness in the cryptocurrency market is more pronounced in the short-run than in the medium- and long-run. BTC, along with LTC and XRP, appears as a leading contributor to the liquidity connectedness in the short run. Contrarily, ETH emerges as a principal contributor to the liquidity connectedness in the long-run analysis. Moreover, frequency-based liquidity clustering analysis shows a tight, short- and long-term clustering compared with the medium-term frequency domain. This evidence provides a possible explanation about the short- and long-term investment preferences of the investors operating in the cryptocurrency market. For example, the tight liquidity clustering points toward investors that hold cryptocurrencies for speculative purposes, whereas long-term clustering indicates that investors use cryptocurrencies as long-term investment assets.

Furthermore, our time-varying analysis shows that liquidity connectedness has increased over time in the cryptocurrency markets. This finding indicates the impact of increasing demand and higher acceptability of this unique asset on the individual and institutional investors in financial markets worldwide. Moreover, short-run liquidity connectedness among cryptocurrencies appears to be more pronounced when analyzing different frequency domains of time-varying connectedness.

Our findings offer several practical implications for cryptocurrency market participants. First, cryptocurrency investors may benefit by adding highly connected currencies in their portfolios during market booms. From another perspective, they can avoid liquidity crunches by diversifying into the least related cryptocurrencies during market busts. Second, we reveal that volatility drives the liquidity connectedness in the cryptocurrency market, as higher connectedness is observed during highly volatile episodes of cryptocurrency prices. This observation indicates that despite its acceptance as a mainstream financial asset, cryptocurrency still serves as a speculative asset and should be cautiously used. Finally, we report that liquidity connectedness is a phenomenon dependent on the time–frequency connectedness that offers diverse opportunities to investors with short- and long-term investment horizons.

Future research may consider exploring the determination of liquidity connectedness in the cryptocurrency market, which can be achieved by either a cross-sectional framework or a dynamic fashion. The cross-sectional study will explain the transmission of liquidity from one cryptocurrency to another. By contrast, the time-varying investigation will uncover the determinants of overall liquidity spillovers across the whole cryptocurrency market.

## Data Availability

Codes and data used for this paper are available upon request.

## Footnotes

1 Liquidity commonality has been rigorously explored for various markets, including bonds, stocks, commodities, and currencies (Chordia et al. 2001; Marshall et al. 2012, 2013; Chuliá et al. 2020; and Inekwe 2020).

2 Although the cryptocurrencies have gained substantial confidence from the investment community, which will have positive consequences for their liquidity, the overall liquidity levels around cryptocurrency market are still much lower than the traditional asset markets. For example, as of January 2020, Bitcoin's monthly trading volume amounted to roughly USD 930 billion compared with USD 1.03 trillion trading volume on the NYSE for the same period, reported by Brauneis et al. (2020).

3 Prior literature also suggests a causal link between liquidity and volatility. For traditional assets, lower liquidity contributes to higher volatility, whereas higher liquidity leads to lower volatility, and vice versa (Będowska-Sójka and Kliber 2019). However, for speculative assets like cryptocurrencies, higher volatility can contribute to higher liquidity (Będowska-Sójka et al. 2020).

4 We do understand that, in line with Bellavitis et al. (2020), regulatory spillovers might affect the quality of liquidity spillovers in cryptocurrency market. However, whether they affect through demand or supply channel, our spillover connectedness measures would take into account these regulatory changes. Nonetheless, an explicit study of such regulatory spillovers’ role is beyond the scope of our work.

5 Foley et al. (2019) report that, as of July 2018, there are over 1800 cryptocurrencies available, whose combined market capitalization exceeded $300 billion. Various online cryptocurrency markets and exchanges trade around $50 billion every day.

6 Kyriazis (2019) also provides a survey of the studies that have thus far investigated the spillovers or connectedness in cryptocurrency market.

7 Diebold and Yilmaz (2012) obtain the variance decompositions through a VAR system which is independent of variable ordering (Koop et al. 1996; Pesaran and Shin 1998). Note that we use the forecast error variance decomposition (FEVD), which is used is the Generalized FEVD of Pesaran and Shin (1998), and not the baseline FEVD.

8 Bitocin, Ethereum and Ripple are top three currencies in term of market capitalization while Litecoin, Monero and Dash stand at 7th, 14th and 19th spot, respectively. Coindesk.com/coindesk20 (accessed on 2020, April 04).

9 As of 9th April 2020, the average transaction time for Ripple transaction is between 3 and 5 s and transaction cost is 0.0000051. See https://ripple.com/xrp/

10 During 2017 Litecoin, Ripple and Bitcoin experienced price appreciation of 5000%, 36,000% and 1300%, respectively. Authors’ calculation based on respective cryptocurrency prices during 2017.

11 Foley et al. (2018) report that there are more than one hundred hedge funds exclusively focusing on bitcoin and related “cryptocurrencies.” Moreover, in late 2017, the Chicago merchantile exchange (CME) launched a bitcoin futures contract in response to demand from traders and hedgers.

## References

[CR1] Ahmad W, Mishra AV, Daly KJ (2018). Financial connectedness of BRICS and global sovereign bond markets. Emerg Mark Rev.

[CR2] Al-Yahyaee KH, Mensi W, Ko HU, Yoon SM, Kang SH (2020). Why cryptocurrency markets are inefficient: the impact of liquidity and volatility. N Am J Econ Financ.

[CR3] Amihud Y (2002). Illiquidity and stock returns: cross-section and time-series effects. J Financ Mark.

[CR4] Antonakakis N, Chatziantoniou I, Gabauer D (2019). Cryptocurrency market contagion: market uncertainty, market complexity, and dynamic portfolios. J Int Financ Mark Inst Money.

[CR5] Balli F, Naeem MA, Shahzad SJH, de Bruin A (2019). Spillover network of commodity uncertainties. Energy Econ.

[CR6] Balli F, de Bruin A, Chowdhury MIH, Naeem MA (2020). Connectedness of cryptocurrencies and prevailing uncertainties. Appl Econ Lett.

[CR7] Baruník J, Kley T (2019). Quantile coherency: a general measure for dependence between cyclical economic variables. Econom J.

[CR8] Baruník J, Křehlík T (2018). Measuring the frequency dynamics of financial connectedness and systemic risk. J Financ Econom.

[CR10] Baruník J, Kočenda E, Vácha L (2017). Asymmetric volatility connectedness on the forex market. J Int Money Financ.

[CR11] Baumöhl E (2019). Are cryptocurrencies connected to forex? A quantile cross-spectral approach. Financ Res Lett.

[CR12] Baur DG, Cahill D, Godfrey K, Liu ZF (2019). Bitcoin time-of-day, day-of-week and month-of-year effects in returns and trading volume. Financ Res Lett.

[CR13] Będowska-Sójka B, Kliber A (2019). The causality between liquidity and volatility in the Polish stock market. Financ Res Lett.

[CR14] Będowska-Sójka B, Hinc T, Kliber A (2020). Volatility and liquidity in cryptocurrency markets—the causality approach. Contemporary trends and challenges in finance.

[CR15] Bellavitis C, Cumming D, Vanacker T (2020) Ban, boom, and echo! Entrepreneurship and initial coin offerings. Entrep Theor Pract 1042258720940114

[CR16] Bodart V, Candelon B (2009). Evidence of interdependence and contagion using a frequency domain framework. Emerg Mark Rev.

[CR17] Borri N, Shakhnov K (2019). Regulation spillovers across cryptocurrency markets. Financ Res Lett.

[CR18] Bouri E, Gupta R, Roubaud D (2019). Herding behaviour in cryptocurrencies. Financ Res Lett.

[CR19] Bouri E, Lucey B, Roubaud D (2019). The volatility surprise of leading cryptocurrencies: transitory and permanent linkages. Financ Res Lett.

[CR20] Bouri E, Roubaud D, Shahzad SJH (2019). Do Bitcoin and other cryptocurrencies jump together?. Q Rev Econ Financ.

[CR21] Bouri E, Shahzad SJH, Roubaud D (2019). Co-explosivity in the cryptocurrency market. Financ Res Lett.

[CR22] Bouri E, Shahzad SJH, Roubaud D (2020). Cryptocurrencies as hedges and safe-havens for US equity sectors. Q Rev Econ Financ.

[CR23] Bouri E, Gabauer D, Gupta R, Tiwari AK (2021). Volatility connectedness of major cryptocurrencies: the role of investor happiness. J Behav Exp Financ.

[CR24] Bouri E, Saeed T, Vo XV, Roubaud D (2021). Quantile connectedness in the cryptocurrency market. J Int Financ Mark Inst Money.

[CR25] Brauneis A, Mestel R, Riordan R, Theissen E (2020) How to measure the liquidity of cryptocurrencies? Available at SSRN

[CR26] Bredin D, Conlon T, Potì V (2017). The price of shelter-Downside risk reduction with precious metals. Int Rev Financ Anal.

[CR27] Brunnermeier MK, Pedersen LH (2009). Market liquidity and funding liquidity. Rev Financ Stud.

[CR28] Caporale GM, Kang WY, Spagnolo F, Spagnolo N (2021). Cyber-attacks, spillovers and contagion in the cryptocurrency markets. J Int Financ Mark Inst Money.

[CR29] Cheung A, Roca E, Su JJ (2015). Crypto-currency bubbles: an application of the Phillips–Shi–Yu (2013) methodology on Mt. Gox Bitcoin Prices Appl Econ.

[CR30] Chordia T, Roll R, Subrahmanyam A (2000). Commonality in liquidity. J Financ Econ.

[CR31] Chordia T, Roll R, Subrahmanyam A (2001). Market liquidity and trading activity. J Financ.

[CR32] Christiansen C (2007). Volatility-spillover effects in European bond markets. Eur Financ Manag.

[CR33] Chuliá H, Koser C, Uribe JM (2020). Uncovering the time-varying relationship between commonality in liquidity and volatility. Int Rev Financ Anal.

[CR34] Corbet S, Lucey B, Yarovaya L (2018). Datestamping the Bitcoin and Ethereum bubbles. Financ Res Lett.

[CR35] Coughenour JF, Saad MM (2004). Common market makers and commonality in liquidity. J Financ Econ.

[CR36] da Gama Silva PVJ, Klotzle MC, Pinto ACF, Gomes LL (2019). Herding behavior and contagion in the cryptocurrency market. J Behav Exp Financ.

[CR37] Diebold FX, Liu L, Yilmaz K (2017) Commodity connectedness (No. w23685). National Bureau of Economic Research

[CR38] Diebold FX, Yilmaz K (2009). Measuring financial asset return and volatility spillovers, with application to global equity markets. Econ J.

[CR39] Diebold FX, Yilmaz K (2012). Better to give than to receive: predictive directional measurement of volatility spillovers. Int J Forecast.

[CR40] Diebold FX, Yılmaz K (2014). On the network topology of variance decompositions: measuring the connectedness of financial firms. J Econom.

[CR41] Diebold FX, Yilmaz K (2015). Trans-Atlantic equity volatility connectedness: US and European financial institutions, 2004–2014. J Financ Econom.

[CR42] Dyhrberg AH, Foley S, Svec J (2018). How investible is Bitcoin? Analyzing the liquidity and transaction costs of Bitcoin markets. Econ Lett.

[CR44] Foley S, Karlsen JR, Putniņš TJ (2019). Sex, drugs, and bitcoin: How much illegal activity is financed through cryptocurrencies?. Rev Financ Stud.

[CR45] Fong KY, Holden CW, Tobek O (2018) Are volatility over volume liquidity proxies useful for global or US research? Kelley School of Business Research Paper (17–49)

[CR46] Fousekis P, Tzaferi D (2021). Returns and volume: frequency connectedness in cryptocurrency markets. Econ Model.

[CR48] Fry J, Cheah ET (2016). Negative bubbles and shocks in cryptocurrency markets. Int Rev Financ Anal.

[CR49] Gençay R, Gradojevic N, Selçuk∥ F, Whitcher B (2010). Asymmetry of information flow between volatilities across time scales. Quant Financ.

[CR50] Gurdgiev C, O'Loughlin D (2020). Herding and anchoring in cryptocurrency markets: Investor reaction to fear and uncertainty. J Behav Exp Financ.

[CR51] Hameed A, Kang W, Viswanathan S (2010). Stock market declines and liquidity. J Financ.

[CR52] Hasan M, Arif M, Naeem MA, Ngo QT, Taghizadeh-Hesary F (2021). Time-frequency connectedness between Asian electricity sectors. Econ Anal Policy.

[CR53] Hasbrouck J, Seppi DJ (2001). Common factors in prices, order flows, and liquidity. J Financ Econ.

[CR54] Hu AS, Parlour CA, Rajan U (2019). Cryptocurrencies: Stylized facts on a new investible instrument. Financ Manag.

[CR55] Huynh TLD, Nasir MA, Vo XV, Nguyen TT (2020). “Small things matter most”: the spillover effects in the cryptocurrency market and gold as a silver bullet. N Am J Econ Financ.

[CR56] Inekwe JN (2020). Liquidity connectedness and output synchronisation. J Int Financ Mark Inst Money.

[CR57] Ji Q, Bouri E, Lau CKM, Roubaud D (2019). Dynamic connectedness and integration in cryptocurrency markets. Int Rev Financ Anal.

[CR58] Kamara A, Lou X, Sadka R (2008). The divergence of liquidity commonality in the cross-section of stocks. J Financ Econ.

[CR59] Katsiampa P, Corbet S, Lucey B (2019). Volatility spillover effects in leading cryptocurrencies: a BEKK-MGARCH analysis. Financ Res Lett.

[CR60] Kim T (2017). On the transaction cost of Bitcoin. Financ Res Lett.

[CR61] Koch A, Ruenzi S, Starks L (2016). Commonality in liquidity: a demand-side explanation. Rev Financ Stud.

[CR62] Koop G, Pesaran MH, Potter SM (1996). Impulse response analysis in nonlinear multivariate models. J Econom.

[CR63] Kou G, Peng Y, Wang G (2014). Evaluation of clustering algorithms for financial risk analysis using MCDM methods. Inf Sci.

[CR64] Kou G, Akdeniz ÖO, Dinçer H, Yüksel S (2021). Fintech investments in European banks: a hybrid IT2 fuzzy multidimensional decision-making approach. Financ Innov.

[CR66] Koutmos D (2018). Liquidity uncertainty and Bitcoin’s market microstructure. Econ Lett.

[CR69] Kyriazis NA (2019). A survey on empirical findings about spillovers in cryptocurrency markets. J Risk Financ Manag.

[CR70] Laurent S, Lecourt C, Palm FC (2016). Testing for jumps in conditionally Gaussian ARMA–GARCH models, a robust approach. Comput Stat Data Anal.

[CR71] Loi H (2018). The liquidity of bitcoin. Int J Econ Financ.

[CR72] Luu Duc Huynh T (2019). Spillover risks on cryptocurrency markets: a look from VAR-SVAR granger causality and student’st copulas. J Risk Financ Manag.

[CR74] Ma F, Zhang Y, Wahab MIM, Lai X (2019). The role of jumps in the agricultural futures market on forecasting stock market volatility: new evidence. J Forecast.

[CR76] Marshall BR, Nguyen NH, Visaltanachoti N (2012). Commodity liquidity measurement and transaction costs. Rev Financ Stud.

[CR77] Marshall BR, Nguyen NH, Visaltanachoti N (2013). Liquidity commonality in commodities. J Bank Financ.

[CR78] Moratis G (2020). Quantifying the spillover effect in the cryptocurrency market. Financ Res Lett.

[CR79] Naeem MA, Farid S, Balli F, Hussain Shahzad SJ (2020). Hedging the downside risk of commodities through cryptocurrencies. Appl Econ Lett.

[CR80] Naeem MA, Mbarki I, Suleman MT, Vo XV, Shahzad SJH (2020). Does Twitter Happiness Sentiment predict cryptocurrency?. Int Rev Financ.

[CR81] Naeem MA, Bouri E, Peng Z, Shahzad SJH, Vo XV (2021). Asymmetric efficiency of cryptocurrencies during COVID19. Phys A Stat Mech Appl.

[CR82] Naeem MA, Mbarki I, Shahzad SJH (2021). Predictive role of online investor sentiment for cryptocurrency market: evidence from happiness and fears. Int Rev Econ Financ.

[CR83] Naeem MA, Qureshi S, Rehman MU, Balli F (2021). COVID-19 and cryptocurrency market: evidence from quantile connectedness. Appl Econ.

[CR84] Omane-Adjepong M, Alagidede IP (2019). Multiresolution analysis and spillovers of major cryptocurrency markets. Res Int Bus Financ.

[CR85] Pesaran HH, Shin Y (1998). Generalized impulse response analysis in linear multivariate models. Econ Lett.

[CR86] Phillips PC, Shi S, Yu J (2015). Testing for multiple bubbles: historical episodes of exuberance and collapse in the S&P 500. Int Econ Rev.

[CR88] Scharnowski S (2021). Understanding bitcoin liquidity. Financ Res Lett.

[CR89] Shahzad SJH, Hernandez JA, Rehman MU, Al-Yahyaee KH, Zakaria M (2018). A global network topology of stock markets: transmitters and receivers of spillover effects. Phys A Stat Mech Appl.

[CR90] Shahzad SJH, Bouri E, Arreola-Hernandez J, Roubaud D, Bekiros S (2019). Spillover across Eurozone credit market sectors and determinants. Appl Econ.

[CR92] Shahzad SJH, Bouri E, Ahmad T, Naeem MA (2021). Extreme tail network analysis of cryptocurrencies and trading strategies. Financ Res Lett.

[CR93] Shahzad SJH, Bouri E, Ahmad T, Naeem MA, Vo XV (2021). The pricing of bad contagion in cryptocurrencies: a four-factor pricing model. Financ Res Lett.

[CR94] Smales LA (2019). Bitcoin as a safe haven: is it even worth considering?. Financ Res Lett.

[CR95] Vidal-Tomás D, Ibáñez AM, Farinós JE (2019). Herding in the cryptocurrency market: CSSD and CSAD approaches. Financ Res Lett.

[CR96] Wei WC (2018). Liquidity and market efficiency in cryptocurrencies. Econ Lett.

[CR97] Xu Q, Zhang Y, Zhang Z (2021). Tail-risk spillovers in cryptocurrency markets. Financ Res Lett.

[CR98] Xu Q, Zhang Y, Zhang Z (2020) Tail-risk spillovers in cryptocurrency markets. Financ Res Lett 101453

[CR99] Yi S, Xu Z, Wang GJ (2018). Volatility connectedness in the cryptocurrency market: Is Bitcoin a dominant cryptocurrency?. Int Rev Financ Anal.

[CR100] Zha Q, Kou G, Zhang H, Liang H, Chen X, Li CC, Dong Y (2020). Opinion dynamics in finance and business: a literature review and research opportunities. Financ Innov.

[CR101] Zięba D, Kokoszczyński R, Śledziewska K (2019). Shock transmission in the cryptocurrency market. Is Bitcoin the most influential?. Int Rev Financ Anal.

